# A nanoporous gold-based electrochemical aptasensor for sensitive detection of cocaine†

**DOI:** 10.1039/c9ra01292c

**Published:** 2019-05-07

**Authors:** Nahid Tavakkoli, Nasrin Soltani, Fatemeh Mohammadi

**Affiliations:** Department of Chemistry, Payame Noor University P. O. Box 19395-3697 Tehran Iran Tavakkolinahid@yahoo.com +98-313-7381002

## Abstract

The increasing application of aptamers in bioassays has triggered a lot of research interest for development of highly sensitive and selective sensing platforms. Herein, we report on the design of a sensitive cocaine biosensor by immobilizing the 5′-disulfide-functionalized end of an aptamer sequence on a nanoporous gold (NPG) electrode followed by the conjugation of its 3′-amino-functionalized end to 2,5-dihydroxybenzoic acid (DHBA) as the redox probe. In the presence of cocaine, the aptamer undergoes a conformational change from an open unfolded state to a closed conformation, which reduces the distance between DHBA and the electrode surface, resulting in the enhanced electron-transfer efficiency. Using square wave voltammetric method and under the optimal conditions, the cocaine aptasensor presented two linear responses in the concentration ranges between 0.05–1 and 1–35 μM, with an excellent detection limit of 21 nM. The proposed aptasensor provides a simple and low-cost method for cocaine detection with good reproducibility and accuracy. Furthermore, it could be regarded as a general model to investigate the unique function of aptamer-functionalized nanostructured electrodes to stablish highly advanced electrochemical biosensors for various target analytes of diagnostic importance.

## Abstract 2

The increasing application of aptamers in bioassays has triggered a lot of research interest for development of highly sensitive and selective sensing platforms.

## Graphical abstract

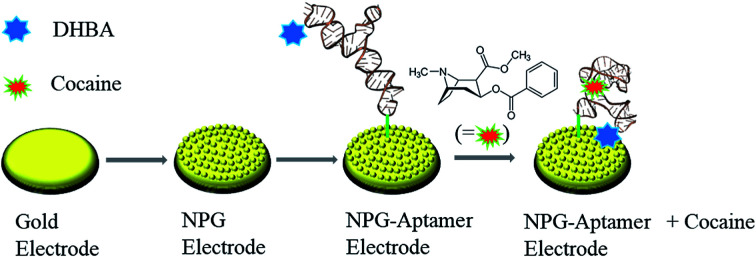

## 1. Introduction

Drug abuse continues to be a growing global concern, causing serious health and economic implications worldwide.^1,2^ Cocaine, also known as benzoylmethylecgonine, is one of the most widely abused drugs all over the world and is known to be a strong stimulant to the central nervous system.^3^ Cocaine abuse can cause many adverse effects on human such as anxiety, heart failure and organ damage.^4,5^ Therefore, development of reliable, sensitive and selective methods for detection of trace amounts of cocaine is of paramount importance. To date, several analytical methods have been developed for determination of cocaine, including high performance liquid chromatography,^6,7^ gas-chromatography-mass spectrometry,^8^ ion mobility spectrometry,^9–11^ surface-enhanced Raman scattering spectroscopy,^12,13^ chemiluminescence,^14^ fluorescence,^15,16^ colorimetry^17,18^ and piezoelectric.^19^ Although most of the mentioned techniques provide satisfactory sensitivities for cocaine detection, but development of simple, cost-effective and fast analytical methods for sensitive and selective detection of cocaine is still an open research area. Electrochemical biosensors have been proved to have the advantages of being inexpensive, easy to use, rapid and robust compared to the other methods for cocaine detection.^20,21^

Aptamers are short single-stranded nucleic acid oligomers that have attracted a lot of research interest, owing to their outstanding and unique properties including small size, ease of synthesis, low immunogenicity, high binding specificity, high resistance against denaturation, stability over wide temperature and pH ranges and fast refolding to the original structure.^22^ In the past two decades, aptamer-based biosensors with high specificity and affinity have become a significant diagnostic tool for capturing and detection of a wide number of analytes ranging from small molecules,^23,24^ ions,^25,26^ peptides,^27^ proteins^28^ and cells.^29^ Nanotechnology has been widely used as an efficient tool to improve the sensitivity of all kinds of sensors and biosensors including aptasensors by signal amplification on a wide variety of nanomaterials and nanostructures such as metal nanoclusters and nanoparticles,^30–32^ electrospun nanofibers,^33,34^ conjugated polymers^35–37^ and carbon-based materials.^38^ The integration of electrochemical detection techniques with aptamer- and nano-technologies is a unique combination, enabling the development of highly advanced sensing platforms for detection of almost all kinds of target analytes in a sensitive, selective, fast and reliable manner.

Herein, a novel nanostructured electrochemical aptasensor was designed and applied for the sensitive and selective detection of cocaine. A nanoporous gold (NPG) electrode was fabricated by anodizing a flat gold electrode followed by treatment with ascorbic acid as reducing reagent and used for the further modification steps. The NPG electrode was then functionalized with aptamer and redox probe to construct the cocaine aptasensor. Effect of important parameters affecting the biosensor response and performance were studied and optimized. It was showed that under the optimal conditions the proposed aptasensor could be effectively used for sensitive and selective detection of cocaine.

## 2. Experimental

### 2.1. Reagents

All reagents were of analytical grade and used as received without further purification. Sodium hydroxide, *n*-hydroxy-succinimide (NHS), 1-ethyl-3-(3-dimethylaminopropyl) carbodiimide hydrochloride (EDC), tris(2-carboxyethyl)phosphine hydrochloride (TCEP), 6-mercapto-1-hexanol (MCH), 2,5-dihydroxybenzoic acid (DHBA), ascorbic acid, caffeine, codeine, heroin, di-sodium hydrogen phosphate, sodium dihydrogen phosphate, potassium dihydrogen phosphate, di-potassium hydrogen phosphate, potassium chloride, sodium chloride, potassium ferricyanide, potassium ferrocyanide, magnesium chloride and calcium chloride were obtained from Sigma-Aldrich (Munich, Germany). A previously reported aptamer,^39^ 5′AGACAAGGAAAATCCTTCAATGAAGTGGGTCG-3′, which is functionalized with disulfide and amine groups at its 5′ and 3′ terminals respectively was purchased from Faza Pajooh Biotech (Iran). Cocaine was obtained from Iran Drug Control Headquarters (Iran). Morphine was obtained from TEMAD Company (Iran). Double-distilled deionized water was used in all solution preparations.

### 2.2. Apparatus

All electrochemical measurements were performed using an Ivium potentiostat/galvanostat (Vertex, Ivium Technologies, Netherlands) with a functionalized gold electrode, Ag/AgCl/3.0 M KCl and a platinum wire, used as the working, reference and auxiliary electrodes, respectively. The surface morphology of the flat and NPG electrodes was investigated on a Hitachi FE-SEM system model S-4160.

### 2.3. Fabrication of NPG electrode

In order to prepare the NPG electrode, a flat gold electrode was immersed in 0.1 M phosphate buffer (pH 7.4) and then was anodized by applying a potential of 3 V for 180 s, resulting in a highly oxidized gold electrode surface. While applying the potential, the electrode was gently tapped to remove the evolved bubbles and keep the conductivity constant. Then, the electrode was immersed in a freshly prepared 1 M ascorbic acid solution for 5 min. Ascorbic acid reduces gold oxide to metallic Au, giving rise to a porous gold surface. Upon the treatment with ascorbic acid, the electrode surface turned dark, which could be attributed to the formation of gold nanocrystals and the subsequent enhancement in the electrode surface area.

### 2.4. Fabrication of the cocaine aptasensor

**Scheme 1:**
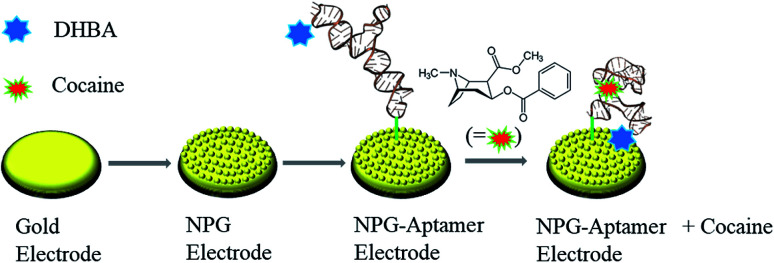
Schematic illustration of the fabrication process of the nanoporous gold-based cocaine aptasensor.

The aptasensor was fabricated by immobilizing cocaine aptamer on the NPG electrode followed by modification with DHBA as the responsive probe. Scheme 1 illustrates different steps for the fabrication of the cocaine aptasensor. The aptamer used in this work is functionalized with both amine and disulfide groups. The disulfide bond of the cocaine aptamer needs to be reduced to thiol (S–H) group prior to its attachment to the gold electrode surface. To achieve this, 40 μL of 4 μM aptamer was mixed with 25 μL of 0.04 mM TCEP and 30 μL of 0.1 M phosphate buffer (pH 7.4) and incubated for 1 h in the dark. Then, 10 μL of the above solution was mixed with 5 μL of 2 M MgCl_2_ and 3 μL of 0.3 M NaH_2_PO_4_. The obtained solution was applied to the electrode surface for 12 h to obtain a self-assembled monolayer of aptamer on the NPG electrode. Subsequently, the NPG electrode was passivated by 4 μM MCH for 6 h to remove nonspecific aptamer adsorption on the electrode surface.

In the next step, the responsive probe, DHBA, was attached to the modified NPG electrode through amide bond formation between amine group (–NH_2_) of the aptamer and carboxyl group (–COOH) of DHBA activated with EDC and NHS. Briefly, 100 μL of DHBA (0.5 mM) containing 20 mM of EDC, 30 mM of NHS and 0.01 M of phosphate buffer (pH 5.5) was applied to the electrode surface for 2 h. Subsequently, the electrode was rinsed with water to remove the excess unreacted reagents.

## 3. Results and discussion

### 3.1. Characterization of the NPG electrode

**Fig. 1:**
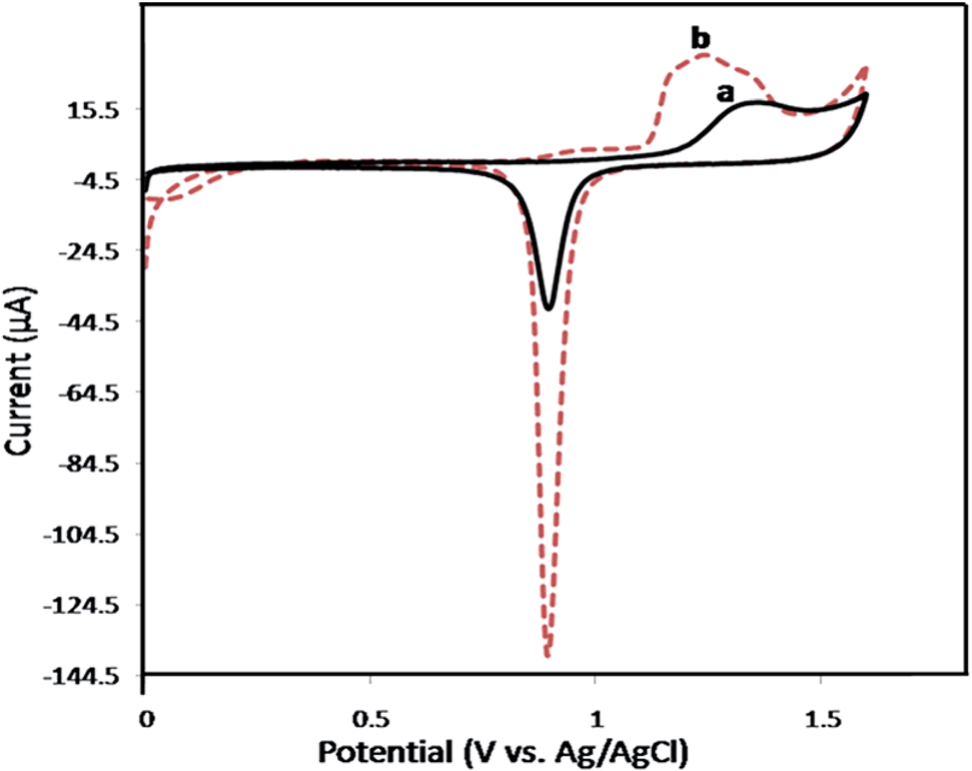
Cyclic voltammograms of the flat gold electrode (a) and NPG electrode (b) at a scan rate of 50 mV s^−1^ in 0.5 M H_2_SO_4_.

Cyclic voltammetry (CV) was used to study the changes in the electrochemically active surface area of gold electrode before and after modification. Fig. 1 shows the cyclic voltammograms of the flat and NPG electrodes in 0.5 M H_2_SO_4_. As seen, the cathodic peak at ∼0.9 V, which is due to the electrochemical reduction of gold oxide to metallic Au shows a considerable increase in the case of NPG electrode, indicating the enhanced electrochemically active sites of the NPG electrode compared to the flat electrode.

The real surface area of the flat gold and NPG electrodes were determined according to the literature by integrating the cathodic peak at 0.9 V and the values were found to be 0.19 ± 0.01 and 0.69 ± 0.02 cm^2^ for the flat and NPG electrodes, respectively.^40^ The real surface area of the NPG electrode is 3.7 times that of flat electrode, suggesting the effective electrode surface processing.

**Fig. 2:**
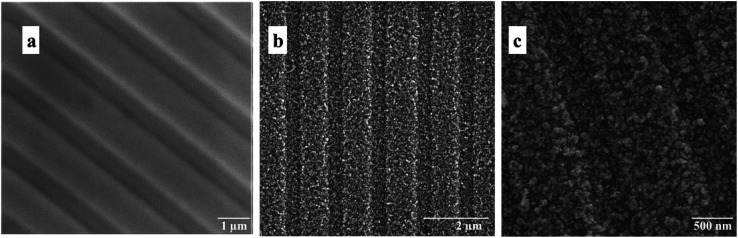
SEM images of the flat (a) and NPG electrode at different magnifications (b and c).

Scanning electron microscopy technique was used to further study and visualize the morphology of the flat and NPG electrodes. As shown in Fig. 2, the flat gold electrode possesses a smooth structure, which changes to a nanoporous form with pore sizes as small as 50 ± 10 nm after surface modification.

### 3.2. Investigation of the aptasensor fabrication process

**Fig. 3:**
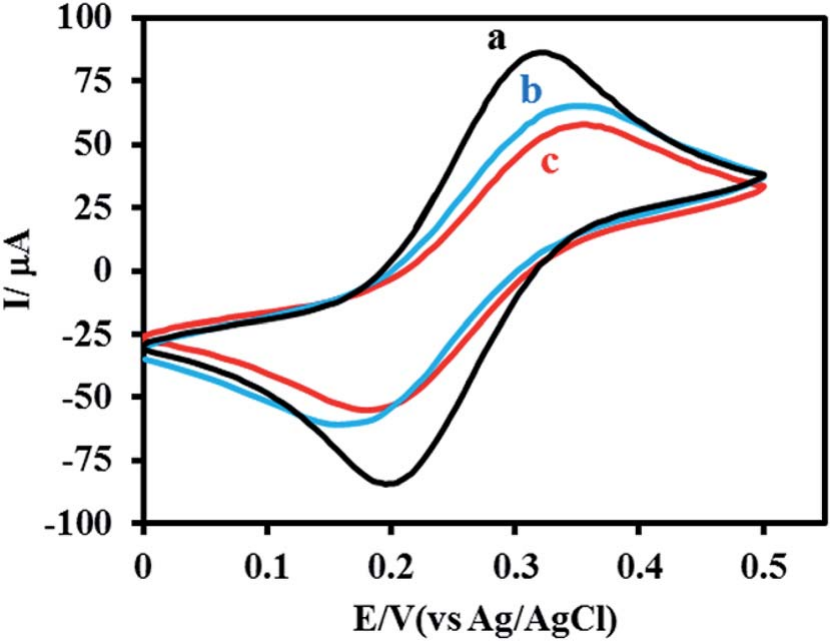
Cyclic voltammograms of 0.5 mM of [Fe(CN)_6_]^3−/4−^ in 0.1 M PBS, pH 5.5, containing 0.1 M KCl at a scan rate of 50 mV s^−1^ obtained for electrodes at different assembly steps: flat gold (a), NPG (b) and aptamer functionalized NPG electrode before (c) and after immobilization with DHBA redox probe.

CV was applied as a marker to study the changes in the electrochemical properties of the electrode upon each assembly step. A phosphate buffer solution (0.1 M, pH 5.5) containing 0.1 M KCl and 0.5 mM [Fe(CN)_6_]^3−/4−^ was used to conduct all cyclic voltammetry measurements. The cyclic voltammograms at different assembly steps are shown in Fig. 3. Upon the immobilization of aptamer on the NPG electrode surface, the peak current decreases while the peak-to-peak separation between the cathodic and anodic waves of the redox probe (Δ*E*_p_) increases. This is due to the development of negative charges on the electrode surface, originating from the phosphate groups of aptamer chain. This indicates that upon the aptamer assembly step, the electron transfer on the electrode surface is limited. Moreover, the steric hindrance on the electrode surface caused by the bulky aptamer is another possible reason for the limited electron transfer.

When DHBA redox probe is assembled at the aptamer-coated electrode surface, the corresponding cyclic voltammogram showed a decrease in peak current and an increase in Δ*E*_p_. The observed changes in the CV plot indicate a further decrease in electron transfer efficiency between [Fe(CN)_6_]^3−/4−^ probe and the electrode surface (Fig. 3, curve c), which could be attributed to the increased steric hindrance on the electrode surface.

**Fig. 4:**
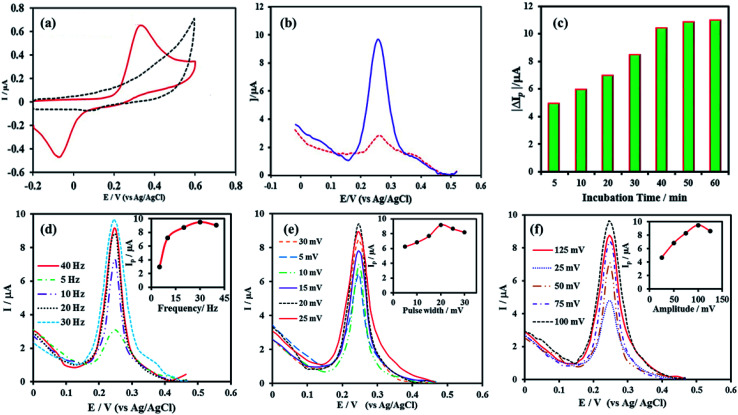
(a) Cyclic voltammograms of the aptamer functionalized NPG electrode in 0.02 M PBS (pH 5.5) at a scan rate of 50 mV s^−1^ before (dashed, black line) and after DHBA immobilization (solid, red line), (b) SWV curves obtained for the proposed aptasensor in 0.02 M PBS, pH 5.5, before (dashed, red line) and after challenging with 1 μM cocaine (solid, blue line), the effect of incubation time (c), frequency (d), pulse width (e) and amplitude (f) on the SWV response of the aptasensor toward cocaine.

The electrochemical activity of the aptasensor was studied using CV in 0.02 M phosphate buffer solution (pH 5.5). The cyclic voltammogram of the electrode shows no oxidation or reduction peaks before DHBA assembly step. After DHBA assembly, it showed well-defined anodic and cathodic peaks, confirming the successful attachment of DHBA redox probe to the amine-functionalized terminal of the aptamer (Fig. 4a). It is worth pointing out that the low peak current of the aptasensor at this step is due the open conformation of aptamer in the absence of cocaine, which causes DHBA redox probe to be in a distant position relative to the NPG electrode surface. It is expected that in the presence of cocaine the aptamer folds into a closed state structure in which the DHBA redox probe is located closer to the electrode surface. This in turn reduces the distance between DHBA and electrode surface and results in the increased efficiency of electron transfer.

### 3.3. Square wave voltammetric determination of cocaine

The sensing performance of the proposed aptasensor toward cocaine was evaluated using square wave voltammetry (SWV) technique. To explore the SWV response of aptasensor to cocaine, the modified electrode was incubated with a 1 μM cocaine solution in 0.02 M phosphate buffer (pH 7.0) containing 1 M NaCl for 40 min. As seen in Fig. 4b, a considerable increase in the SWV peak current is observed upon challenging the aptasensor with cocaine, which confirms the potential application of aptasensor toward cocaine sensing.

In order to obtain the maximum current response for cocaine, SWV parameters (including frequency, step potential and amplitude) and the incubation time of aptasensor with cocaine were investigated and optimized using 10 μM cocaine solution in 0.02 M phosphate buffer (pH 5.5). Since the both aptamer and cocaine structures are sensitive to pH, all electrochemical optimizations were performed in biological pH range (5.5–7.4). The effect of electrode incubation time with cocaine was optimized by immersing the aptasensor in a 10 μM cocaine solution for 5–60 min and then measuring the SWV response (Fig. 4c). As seen, the response current increased up to 40 min and after that became constant. Therefore, an incubation time of 40 min was selected for subsequent studies. The effect of frequency on the SWV response was studied in the range of 5–40. As Fig. 4d shows, the SWV response increased from 5 to 30 Hz and thereafter slightly decreased. Thus, the optimum frequency of 30 Hz was used for the further experiments. An additional parameter that affects the SWV signal is the pulse width. Hence, the effect of pulse width on the response current was investigated in the range between 5–30 mV and the results are presented in Fig. 4e. As the results show the aptasensor gives the most intense SWV response at the pulse width of 20, which was chosen as the optimum value. Effect of potential amplitude on the response current was evaluated by measuring the SWV signal of the aptasensor to cocaine in the amplitude range of 25–125 mV. According to Fig. 4f, 100 mV of amplitude was chosen as the optimum value.

### 3.4. Analytical characterization of the aptasensor

**Fig. 5:**
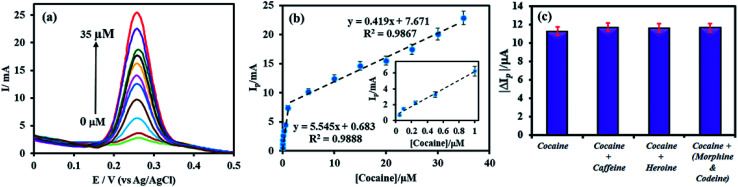
(a) The SWV curves of the aptasensor obtained for different concentrations of cocaine in 0.02 M PBS, pH 5.5, and (b) the corresponding calibration graph of SWV response *versus* cocaine concentration. Inset shows the calibration graph in the concentration range of 0.05–1.00 μM (c) the selectivity of electrochemical aptasensor incubated with 10 μM cocaine in the absence and presence of 100 μM caffeine, heroine and morphine–codeine mixture.

SWV is one of the most sensitive techniques used to study the analytical performance of electrochemical sensors. Hence, the analytical performance of the proposed aptasensor toward cocaine detection was investigated by SWV under optimal conditions. Fig. 5a shows the SWV curves of the aptasensor obtained for different concentrations of cocaine. As can be seen, the SWV peak current increased with increasing the cocaine concentration. The calibration plot of the aptasensor for cocaine indicates two linear ranges over the concentrations of 0.05–1 and 1–35 μM with correlation coefficients (*R*^2^) of 0.9888 and 0.9867, respectively (Fig. 5b). The detection limit of the aptasensor, which was calculated as the amount of cocaine giving a reading equal to three times the standard deviation of background signal, 3*σ* blank, was estimated as follows:
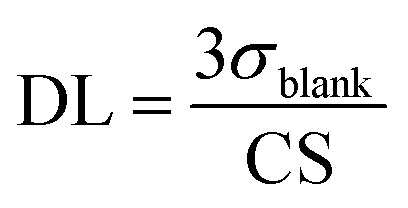


The detection limit was calculated to be 21 nM.

Table 1 compares the response characteristics of the proposed method with the previous cocaine aptasensors. The linear range and detection limit of our method are comparable and even better than most of the existing cocaine aptasensors.

**Table 1:** Comparison of analytical performance of the proposed aptasensor and other aptamer-based sensors in determination of cocaine

Method	Linear range (μM)	Detection limit	Reference
Label free EIS-based aptasensor	0.1–20	100 nM	41
DDIAS^a^-based aptasensor	0.01–5	10 nM	42
Electromagnetic piezoelectric acoustic aptasensor	—	0.3 μM	43
Label-free aptamer-fluorophore assembly	0.2–8	200 nM	44
Enzyme linked aptamer assay	0.1–50	20 nM	45
DNA nanotechnology-based sensing platform	0.1–1000	33 nM	46
Fluorescence anisotropy assay	—	5 μM	47
NPG electrode-based aptasensor	0.05–1 and 1–35	21 nM	This work

a DNA-directed immobilization of aptamer sensors.

Repeatability and reproducibility of the aptasensor were evaluated by SWV measurements of a 10 μM cocaine solution, under optimum experimental conditions. A relative standard deviation (%RSD) of 4.4% was obtained for 5 consecutive scans with the same electrode (Fig. S1†). The reproducibility was evaluated by the measurement of 10 μM cocaine solutions with 5 independently modified electrodes. The relative standard deviation was found to be 4.85% (Fig. S2†). The obtained results confirm the acceptable repeatability and reproducibility of the proposed aptasensor toward cocaine detection. If kept in the freezer (at −20 °C) under nitrogen, the sensor is stable over months.

In order to study the selectivity of the aptasensor, its response to a pure cocaine solution (10 μM) was compared to other cocaine solutions (10 μM) containing caffeine, heroine and morphine–codeine. The concentration of all interferences were 10 times that of cocaine. The changes in SWV signal were found to be 3.6%, 3.9% and 3.2% for caffeine, heroine and morphine–codeine, respectively, indicating the excellent selectivity of aptasensor for cocaine detection.

## 4. Conclusions

In summary, for the first time, we have proposed a highly sensitive and selective aptamer-based biosensing platform on a NPG electrode for square wave voltammetric determination of cocaine traces. The recognition layer was formed by immobilization of the aptamer on the NPG electrode surface followed by the assembly of the DHBA as the redox probe. When cocaine binds to the aptamer, the aptamer undergoes a conformational transition from an open unfolded state to a closed folded one, which results in a considerable enhancement in the electron-transfer efficiency. The proposed cocaine aptasensor exhibits two linear ranges over the concentration ranges of 0.05–1 and 1–35 μM, with a detection limit of 21 nM. Furthermore, this aptasensor provides a simple, low-cost, and accurate method for cocaine detection.

## Conflicts of interest

There are no conflicts to declare.

## Supplementary Material

RA-009-C9RA01292C-s001
