# Supplementary material for: A nanoporous gold-based electrochemical aptasensor for sensitive detection of cocaine
Source: RSC Adv. 2019 May 7;9(25):14296–301. doi: 10.1039/c9ra01292c (PMC9066177; doi:10.1039/c9ra01292c)
Supplement: RA-009-C9RA01292C-s001 [file RA-009-C9RA01292C-s001.pdf]

Electronic Supplementary Material (ESI) for RSC Advances.
This journal is © The Royal Society of Chemistry 2019

Supporting information for:

A nanoporous gold-based electrochemical aptasensor for sensitive detection of cocaine

Department of Chemistry, Payame Noor University, P.O. Box 19395-4697, Tehran, Iran

Corresponding author Email: Tavakkolinahid@yahoo.com

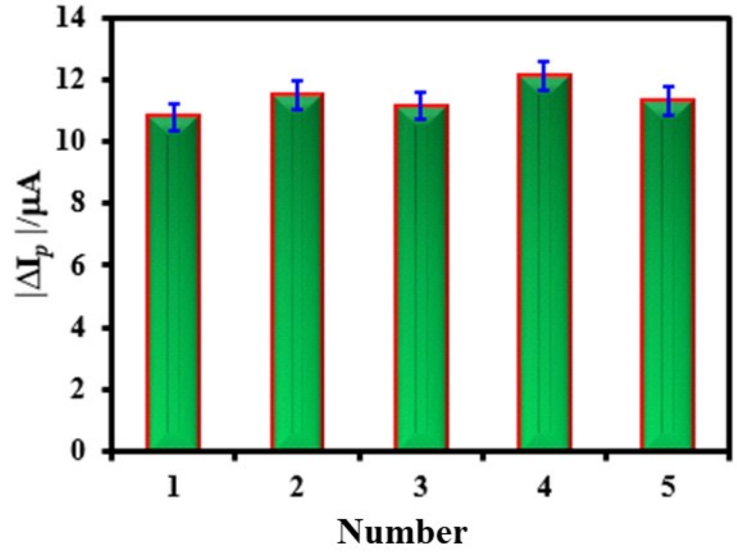

Fig. S1. SWV response of the aptasensor for 5 consecutive scans with the same electrode in 10 μM cocaine solution.

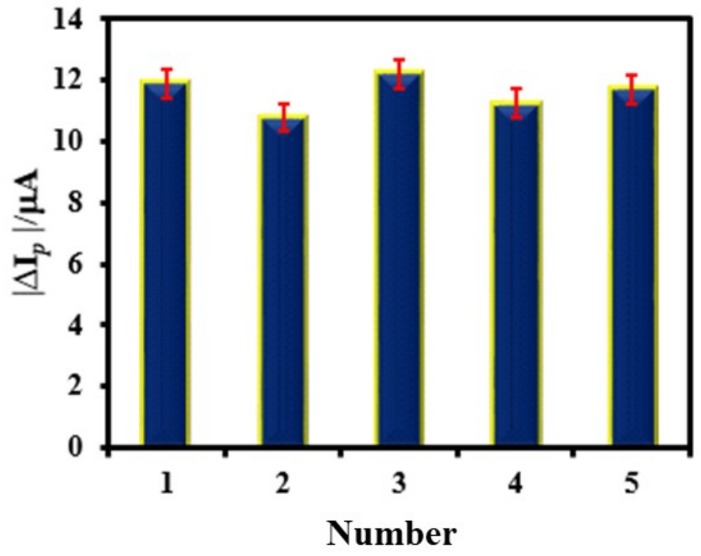

Fig. S2. SWV response of 5 independently modified electrodes toward 10 μM cocaine.
